# Competency in Chest Radiography Interpretation by Junior Doctors and Final Year Medical Students at a Teaching Hospital

**DOI:** 10.1155/2020/8861206

**Published:** 2020-11-06

**Authors:** Bashiru Babatunde Jimah, Anthony Baffour Appiah, Benjamin Dabo Sarkodie, Dorothea Anim

**Affiliations:** ^1^Department of Medical Imaging, School of Medical Sciences, University of Cape Coast, Cape Coast, Ghana; ^2^Ghana Field Epidemiology and Laboratory Training Programme, University of Ghana, Accra, Ghana; ^3^Department of Radiology, University of Ghana, School of Medicine Sciences, Accra, Ghana; ^4^Department of Radiology, Korle Bu Teaching Hospital, Accra, Ghana

## Abstract

**Background:**

Chest radiography (CXR) is a widely used imaging technique for assessing various chest conditions; however, little is known on the medical doctors' and medical students' level of skills to interpret the CXRs. This study assessed the residents, medical officers, house officers, and final year medical students' competency in CXRs interpretation and how the patient's clinical history influences the interpretation.

**Methods:**

We conducted a cross-sectional study in the Cape Coast Teaching Hospital in the Central Region of Ghana among 99 nonradiologists, comprising 10 doctors in residency programmes, 18 medical officers, 33 house officers, and 38 final year medical students. The data collection was done with a semistructured questionnaire in two phases. In phase 1, ten CXRs were presented without patient's clinical history. Phase 2 involved the same ten CXRs presented in the same order alongside the patient's clinical history. Participants were given 3 minutes to interpret each image. Median and interquartile ranges were used to describe continuous variables, while frequencies and percentages were used to describe categorical variables. Test of significant difference and association was conducted using a Wilcoxon rank-sum test/Kruskal–Wallis test and chi-square (*X*^2^) test, respectively.

**Results:**

The average score for interpreting CXRs was 7.0 (IQR = 5–8) and 4.0 (IQR = 3-4), when CXRs were, respectively, presented with and without clinical history. No significant difference was seen in average scores regarding the levels of formal training. Without clinical history, only 40.0% of residents, 22.2% of medical officers, 24.2% of house officers, and 13.2% of medical students correctly interpreted CXRs, while more than 75% each of all categories correctly interpreted CXRs when presented with clinical history. However, all participants had difficulties in identifying CXR with pneumothorax (27.3% vs. 30.3%), pneumomediastinum or left rib fracture (8.1% vs. 33.3%), and lung collapse (37.4% vs. 37.4%) in both situations, with and without patient clinical history.

**Conclusion:**

The patient's clinical history was found to greatly influence doctors' competence in interpreting CXRs. We found a gap in doctors' and medical students' ability to interpret CXRs; hence, the development of this skill should be improved at all levels of medical training.

## 1. Introduction

Evidence-based practice highlights the use of the best available evidence when making clinical decisions about individual patient care [1]. As cited by Andersson et al., “evidence-based medicine is the conscientious, explicit, and judicious use of current best evidence in making decisions about the care of the individual patient” [1]. Medical practice in the tertiary health facility required extensive diversity of skills and competence among various cadre of medical personnel. Medical imaging especially chest radiography is often the first investigation for chest complaints, and the information obtained is key to inform a safe, efficient, and cost-effective intervention for the patients [1–3]. Chest radiography imaging is essential for the diagnosis of several diseases and nonchest-related conditions such as bowel perforation and preoperative assessments of patients [4]. Radiologists are involved in the interpretation of images obtained from computed tomography, magnetic resonance imaging, radiography, ultrasound scan, and others in tertiary hospitals. The inadequate number of radiologists especially in Ghana could hinder a thorough assessment to the overwhelming number of chest radiographs (CXRs). As part of clinical medical training, medical students and junior doctors are endowed with the basic skills to interpret simple radiologic images. However, studies elsewhere indicate poor CXR interpretation skills among medical students and junior doctors [4–7].

Chest radiograph interpretation is less studied worldwide. Miranda et al. assessed the radiological imaging interpretation skills of medical interns in the city of Recife, Pernambuco, Brazil. They observed that both medical interns and final year medical students alike are largely limited in their ability to make radiological diagnoses of simple and commonplace situations [4]. In another study, Christiansen et al. found that Danish junior doctors do not meet the established minimum requirements for radiological diagnostic skills for the use of CXR [7]. These studies suggested formal training for doctors to enhance their skills in interpreting simple radiologic images of their patients. Our search found no published evidence on the competence among various grades of medical doctors and medical students in Ghana. Moreover, this evidence is needed in Ghana to help target interventions among medical doctors in order to maximize the benefit of radiological exams, which is an integral component of our health care delivery. This study, therefore, investigated the competence of residents, medical officers, house officers, and final year medical students in relation to the interpretation of CXR. The study defines a house officer as “a doctor undergoing two-year internship postmedical school,” medical officer as “a doctor who has successfully completed two-year internship postmedical school in cape coast teaching hospital,” and resident as “a medical officer in residency training in cape coast teaching hospital.” This study would provide preliminary data for further assessment and inform effective training of medical practitioners in the interpretation of radiologic images in Ghana.

## 2. Materials and Methods

### 2.1. Study Design

We conducted a cross-sectional study in the Cape Coast Teaching Hospital (CCTH) in the Central Region, Ghana, among 99 nonradiologists. The CCTH is a 400-bed teaching hospital with multispecialty departments and serves as main referral Centre for health Facilities in the Central Region and parts of neighboring regions. The study was conducted between the period of April and May 2018. The nonradiologist medical staffs were purposefully sampled, and they comprise 10 residents, 18 medical officers, 33 house officers, and 38 final year medical students of the University Cape Coast Medical School.

### 2.2. Competence Evaluation

**Figure 1:**
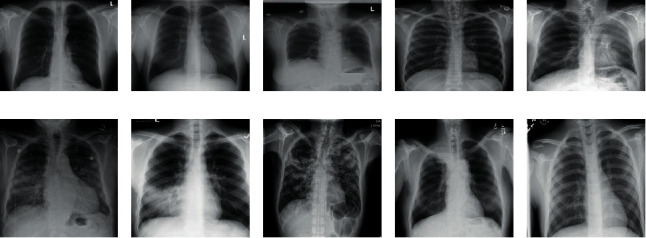
Chest radiographs of patients used for the assessment of competence. The expected diagnoses in line with the specialists' interpretations were (a) pneumothorax, (b) normal image, (c) pneumoperitoneum, (d) pneumomediastinum/left rib fracture, (e) lung collapse, (f) pulmonary edema/congestive cardiac failure, (g) pneumonia, (h) tuberculosis/pneumoconiosis/metastasis, (i) Pancoast tumour/*tuberculosis*, and (j) clavicular fracture/acromioclavicular dislocation.

During the study period, one of the investigators was responsible for administering the test to the participants, in small sessions. A semistructured questionnaire contained the professional rank, participation in formal training on radiograph interpretation, and 10 columns for CXR interpretation. Data collection was conducted in two phases. In the first phase, the participants were presented with 10 CXRs without patients' clinical history one at a time, with a time limit of 3 minutes to interpret each image, and to document their interpretations on the questionnaire (Figure 1). Similar to the first phase, the same 10 CXRs were presented to participants in the same order in the second phase with the clinical history of the patients. Interpretations per slide at each phase were marked and scored as a correct answer or wrong answer, the correct answer was scored 1, and the wrong answer was scored 0. The total score was 10 for each phase, and the score ≥5 was considered pass while <5 was rated as fail.

### 2.3. Data Analysis

Statistical Package for the Social Science (SPSS; IBM Corp, Armonk, NY) version 21 was used to analyze data. The continuous variable was skewed (non-normality distributed) using the Shapiro–Wilk test. Hence, median and interquartile ranges were used to describe the continuous variable, while the Wilcoxon rank-sum test (two levels) and Kruskal–Wallis test (three or more levels) were used to test for significant differences in median scores among groups. Frequencies and percentages were used to describe categorical variables, and comparison of categorical data between subgroups was carried out using the chi-square (*X*^2^) test. All statistical analyses were considered significant at *p* value ≤0.05.

## 3. Results

**Figure 2:**
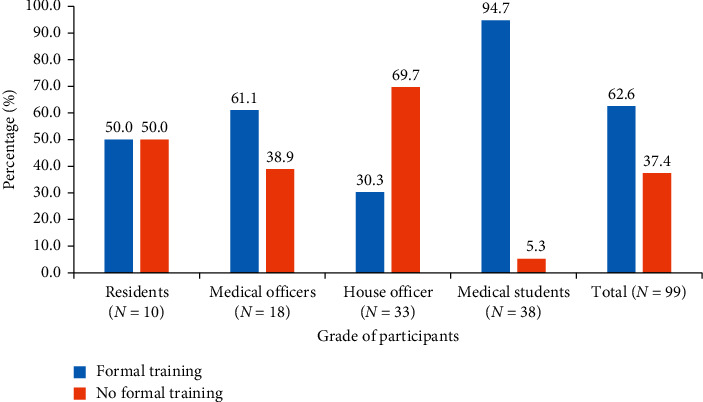
Distribution of participants by grade of profession and participation in formal training on interpreting CXRs.

Of the total of 99 participants, 10 were residents, 18 were medical officers, 33 were house officers, and 38 were final year medical students. Majority (62.6%) of the respondents have participated in formal chest radiography training except house officers (30.3%) (Figure 2). There was significant association between the professional rank of participants and their participation in formal training (chi^2^ (3) = 32.16, *p* < 0.001).

The median score of CXRs interpretation was 4.0 (IQR = 3-4) with no clinical history and 7.0 (IQR = 5–8) when images were presented with clinical history. Without clinical history, nearly equal median scores were recorded by residents (4.0, IQR = 3–5), medical officers (4.0, IQR = 3-4), and house officer (4.0, IQR = 3-4). The median scores increased across the board when clinical history was presented with CXRs with residents scoring the highest (7.5, IQR = 5–9). Generally, a medical student had the least median score with (5.5, IQR = 5–7) and without (3.0, IQR = 2–4) clinical history. The Kruskal–Wallis test shows a significant difference in median scores obtained by participants when presented with clinical history (*p*=0.006). Participants with formal training had lower median scores without any clinical history (3.0, IQR = 3-4) but better when presented with patient's clinical history (7.0, IQR = 5–8) (Table 1).

**Figure 3:**
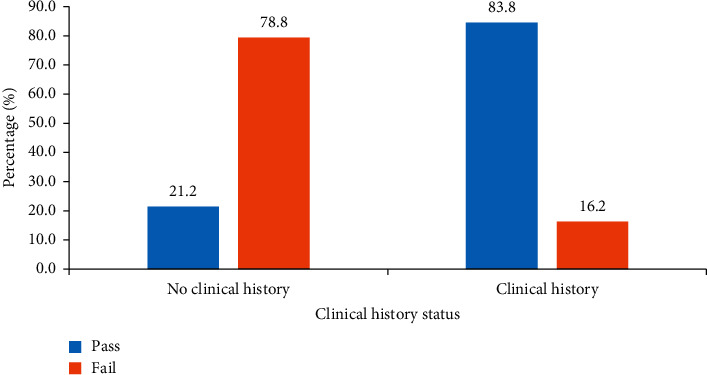
General performance of participant with or without clinical history.

As shown in Figure 3, majority of the participants correctly interpreted the CXRs when presented with clinical history (83.8%) of patients than those without clinical history (21.2%).

The pass rate for CXRs interpretation was much higher with the presentation of patient clinical history. There was no statistically significant association between participants' performance and their professional rank and participation in formal training (Table 2).

With or without clinical history, few of the participants correctly identified CXR with pneumothorax (30.3% vs. 27.3%), pneumomediastinum/left rib fracture (33.3 vs. 8.1%), and lung collapse (37.4% vs. 37.4%). Only 10% of residents, 16.7% of medical officers, 9.1% of house officer, and 15.8% medical students could correctly identify normal radiographs without clinical history. On the contrary, majority of participants could easily identify CXR with pneumonia (64.7% vs. 92.9%) and tuberculosis/pneumoconiosis (64.7% vs. 83.8%). Correct interpretation of CXR with pneumothorax was significantly higher among house officers and residents when CXR was, respectively, presented without (42.4%, *p*=0.037) and with (60%, *p* < 0.001) clinical history. Similarly, correct interpretation of CXR with pneumoperitoneum was significantly higher among residents and medical officers when images were presented without (60%, *p*=0.009) and with (88.9%, *p*=0.037) clinical history, respectively. The medical officers tend to interpret radiographs with clavicular fracture/acromioclavicular dislocation (55.6%) much better than other counterparts (*p*=0.022) in the absence of clinical history (Table 3).

## 4. Discussion

The medical practitioners' ability and competence of interpreting CXRs are vital for patient care [8]. The present study showed that majority (62.6%) of the participants have had formal chest radiography interpretation training. The highest of them was observed among final year medical students (94.7%), while the least was found among house officers (30.3%). At the time of the study, the medical students were in their final stages of clinical training prior to graduation which included chest radiograph interpretation, a possible explanation for the aforementioned. Also, it is entirely possible that many of the house officers trained from institutions outside our studied institution do not recognize their training as formal, rather as a routine lecture. The findings further showed that the professional rank of participants was significantly linked with formal training (*p* < 0.001). This may be misinterpreted that one is likely to be trained on radiographic interpretation as they move higher in their professional rank, but the reverse was seen in this study. As majority of the medical students and medical officers had received formal training compared to residents, house officers were outliners.

Previous studies have shown that training students in radiological imaging interpretation significantly improves their skills and competence on interpreting CXRs [6, 9]. In spite of this, the findings of the present study found no statistically significant association between participants' competence and formal training in interpreting radiographic images. The results show that the average scores for CXRs interpretation was the highest among the residents with (7.5, IQR = 5–9) and without (4.0, IQR = 3–5) clinical history, but mean and median scores did not increase significantly with the level of medical training. Previously, we found that most of the medical students have received some form of radiology training, but they performed the least in CXR interpretation with a median score of 5.5 (IQR = 5–7) (with clinical history) and 3.0 (IQR = 2–4) (without clinical history). In contrast, Eisen et al. [6] found that the median overall score increased with the level of training. The observed differences in findings could be due to the focus of the radiology curriculum, effectiveness of training, and the level of integration in the medical training [9–11]. If training on radiographic interpretation is not effective, CXR interpretation for patients in the outpatient department and emergency will be difficult for medical practitioners. There is the need to focus the radiology course during medical training as well as routine refresher training to improve doctor's confidence and competence [6, 7, 12].

In this study, the competence of residents, medical officers, house officers, and final year medical students in interpreting CXRs heightened the outmost importance of patient's clinical history for doctors to correctly interpret CXRs. For instance, the median score for interpreting CXRs was 4.0 (IQR = 3-4) when no clinical history was presented and 7.5 (IQR = 5–8) when CXRS were presented with clinical history. The observed difference in the median was nearly 2 times when patient's clinical history was available. On the proportion who passed, majority of the participants correctly interpret the CXRs when presented with clinical history (83.8%) while 4 times less was seen when no clinical history (21.2%) was indicated. These findings highlight the relevance of clinical history documentation in medical imaging as well as the physician in interpreting CXRs of their patients. It is, therefore, vital that requesting physicians provide accurate and adequate patient's clinical history when requesting radiographs.

In many teaching hospitals, the frontline staff are predominantly house officers and medical officers. They are expected to request and interpret CXRs and make clinical decisions prior to consultants or senior doctors review. This is particularly important for emergency chest radiographs such as pneumothorax, pneumoperitoneum, pneumomediastinum, lung collapse, pulmonary edema or congestive cardiac failure, pneumonia, *tuberculosis*, clavicular fracture, and acromioclavicular dislocation. The findings indicated that junior doctors and final year medical students alike are limited in their ability to make radiological diagnoses of simple CXRs usually in the absence of clinical history. For instance, both junior doctors and students had difficulties in identifying CXRs with pneumothorax (30.3% vs. 27.3%), pneumomediastinum (33.3% vs. 8.1%), and lung collapse (37.4% vs. 37.4%) in both situations, i.e., with and without patient clinical history. Pneumothorax and rib fractures are somewhat a very common presentation in the emergency room of this tertiary hospital where this study was conducted. It was the expectation that majority of our doctors will know from experience. Similarly, the medical students performed poorly as only 15.8% could correctly identify normal CXR without being presented with clinical history. A significant limitation of the medical interns and students in relation to competency to interpret similar CXRs was reported by Eisen et al. [6] and Miranda et al. [4]. Our findings suggest that more than 85% of the participants had difficulty in interpreting normal CXRs when no clinical history was presented. The tendency that medical doctors and students would interpret the normal CXR as abnormal has been documented by other investigators [4, 6, 7, 13]. Possibly, interpreting a normal CXR as abnormal could lead to inappropriate decisions which might affect the patient's health and unwarranted cost of treatment.

On the other hand, CXRs with pneumonia (64.7% vs. 92.9%) and tuberculosis (64.7% vs. 83.8%) could easily be identified by the majority of our participants. By stratification, correct interpretation of CXR with pneumothorax was significantly higher among house officers (42.4%) and residents (60%), respectively, when CXR was presented without and with clinical history. Similarly, correct interpretation of CXR with pneumoperitoneum was significantly higher among residents (60%) and medical officers (88.9%) when the images were presented without and with clinical history, respectively. The medical officers tend to interpret CXR with clavicular fracture or acromioclavicular dislocation (55.6%) much better than other counterpart in absence of clinical history. The higher level of competence with interpreting CXRs with pneumonia, tuberculosis, pneumothorax, and clavicular fracture could be attributed to experience in practice and not because they had higher training [7]. Therefore, there is the need to intensify refresher training and coaching for all medical doctors to improve their competence.

The findings of the present study could not be generalized to all health facilities in Ghana since it was conducted in a single tertiary health institution and among few medical practitioners. However, to the best of our knowledge, this is the first-time residents, medical officers, house officers, and final year medical students in Ghana have been evaluated in terms of their competence in interpreting CXRs. This study provides preliminary data for further assessment and to inform scale-up training of medical practitioners and students in the interpretation of radiologic images at the Ghana College of Physicians and Surgeons and Medical schools in Ghana.

The present study is probably the first study of CXR interpretation in Ghana and in terms of categories of medical staff involved. Secondly, participants were from multiple fields of medicine and surgery. Thirdly, this study compared participant performance in two different scenarios (with and without clinical history) that directly confirm the importance of clinical history in medical practice, especially in CXR interpretation.

Meanwhile, our study had some limitations: foremost, a small and somewhat arbitrary sample of CXRs was chosen for the survey which does not represent numerous chest anomalies seen in medical practice and while these were representative of common conditions, and results may have been different with other CXRs. Also, there are over 150 cadre of clinicians and 60 final year medical students working at the CCTH, but only 61 clinicians and 38 final year medical students agreed to participate. Hence, we could not generalize the findings from this study.

## 5. Conclusion

This study assessed the competence of residents, medical officers, house officers, and final year medical students in relation to the interpretation of CXRs. The competence in interpreting CXRs of common presentations was moderate in residents, medical officers, and house officers but low among final medical students. The presentation of patient's clinical history was found to significantly improve doctors' competence in interpreting CXRs. We found a gap in their ability to interpret CXRs, and hence, development of this skill should be improved. We recommend that medical training and health training institutions in Ghana could attach importance to the development of effective curricular interventions and reliable assessment methods for radiograph interpretation skills. We also recommend a scale-up assessment of clinicians, radiographers, and medical students CXR interpretation skills to help drive timely interventions in our practice.

## Figures and Tables

**Table 1:** Summary of CXR interpretation score by grade and formal training.

	Frequency	Overall participants' score (out of 10)			
		No clinical history		Clinical history	
		Median	IQR	Median	IQR
*Grade*					
Resident	10	4.0	3–5	7.5	5–9
Medical officer	18	4.0	3-4	7.0	6–8
House officer	33	4.0	3-4	7.0	6–8
Medical student	38	3.0	2–4	5.5	5–7
Total	99	4.0	3-4	7.0	5–8
Kruskal–Wallis test		*X* ^2^ = 6.647, *p*=0.084		*X* ^2^ = 12.448, *p*=0.006	
*Formal training*					
Yes	62	3.0	3-4	7.0	5–8
No	37	4.0	3–5	6.0	6–8
Total	99	4.0	3-4	7.0	5–8
Wilcoxon rank-sum test		*Z* = −1.322, *p*=0.186		*Z* = −0.024, *p*=0.981	

IQR, interquartile range.

**Table 2:** Association between the grade of respondents and formal training and CXRs interpretation score.

Variable	No clinical history			Clinical history		
	Pass	Fail	Chi-square *p* value	Pass	Fail	Chi-square *p* value
*Grade*						
Resident	4 (40.0)	6 (60.0)	0.286	8 (80.0)	2 (20.0)	0.679
Medical officer	4 (22.2)	14 (77.8)		16 (88.9)	2 (11.1)	
House officer	8 (24.2)	25 (75.8)		29 (87.9)	4 (12.1)	
Medical student	5 (13.2)	33 (86.8)		30 (78.0)	8 (12.1)	
Total	21 (21.2)	78 (78.8)		83 (83.8)	16 (16.2)	
*Formal training*						
Yes	11 (17.7)	51 (82.3)	0.274	61 (84.7)	11 (15.3)	0.696
No	10 (27.0)	27 (73.0)		22 (81.5)	5 (13.5)	
Total	21 (21.2)	78 (78.8)		83 (83.8)	16 (16.2)	

**Table 3:** Proportion of correct CXR interpretation by grade of participants.

Slide number, interpretation	Clinical history	Grade of participants (% correct)					
		Residents (*N* = 10)	Medical officers (*N* = 18)	House officer (*N* = 33)	Medical students (*N* = 38)	Total (*N* = 99)	Chi-square *p* value
1, pneumothorax	No	40.0	11.1	42.4	18.4	27.3	0.037^*∗*^
	Yes	60.0	5.6	57.6	10.5	30.3	<0.001^*∗∗*^
2, normal image	No	10.0	16.7	9.1	15.8	13.1	0.803
	Yes	70.0	66.7	78.8	57.9	67.7	0.313
3, pneumoperitoneum	No	60.0	27.8	21.2	10.5	22.2	0.009^*∗*^
	Yes	80.0	88.9	81.8	57.9	73.7	0.039^*∗*^
4, pneumomediastinum	No	10.0	5.6	6.1	10.5	8.1	0.875
	Yes	50.0	55.6	24.2	26.3	33.3	0.062
5, lung collapse	No	30.0	33.3	54.6	26.3	37.4	0.090
	Yes	30.0	44.4	42.4	31.6	37.4	0.677
6, pulmonary edema	No	40.0	38.9	51.5	47.4	46.5	0.817
	Yes	90.0	88.9	81.8	89.3	86.9	0.848
7, pneumonia	No	70.0	72.2	60.6	63.2	64.7	0.837
	Yes	100.0	94.4	84.9	97.4	92.9	0.155
8, tuberculosis	No	70.0	72.2	75.8	50.0	64.7	0.115
	Yes	90.0	100.0	84.9	73.7	83.8	0.083
9, Pancoast tumour	No	50.0	22.2	48.5	42.1	41.4	0.293
	Yes	60.0	61.1	75.8	47.4	60.6	0.113
10, clavicular fracture	No	30.0	55.6	15.2	39.5	33.3	0.022^*∗*^
	Yes	50.0	66.7	72.7	65.8	66.7	0.613

^*∗∗*^significant at *p*-value < 0.001^*∗*^significant at p-value *p*-value < 0.05.

## Data Availability

The data used to support the findings of this study are included within the article.
